# Supplementary material for: SARS-CoV-2 receptor binding domain fusion protein efficiently neutralizes virus infection
Source: PLoS Pathog. 2021 Dec 20;17(12):e1010175. doi: 10.1371/journal.ppat.1010175 (PMC8722722; doi:10.1371/journal.ppat.1010175)
Supplement: Attachment — Submitted filename: PbP.docx [file ppat.1010175.s001.docx]

**Part I - Summary**

Reviewer #1:

Comment: The authors have explored the very well-known interaction between the SARS-CoV-2 RBD and human ACE2 and the potential to block this interaction, demonstrating what others have previously shown – those soluble dimeric forms of these proteins can inhibit SARS-CoV-2 infection vitro and in vivo. The activity they observe is clear, but relatively inefficient and does not compare well with many of the highly potent antibody therapies that achieve the same goal. Indeed, a dose of 75ug RBD-dimer per mouse was only partially effective at preventing infection (50% survival of mice at this dose) and the authors did not explore higher doses to see if prevention of infection was even possible with these reagents.

Answer: We thank the reviewer for the critical reading of our manuscript. We have responded to all comments and amended the manuscript accordingly (changes are in track changes).

As far as we know it has not been previously shown that RBD-Ig can block interaction in-vivo and RBD-Ig was only use as a vaccine and not as therapeutic agent as we show here. We now mention this in the discussion section.

Furthermore, the existing therapies such as highly potent antibodies target the spike protein, which is constantly mutating, as we observe with the emerging variants, rendering such treatments less efficient. We now mention this in the introduction and in the discussion section. In contrast, we use here an entire domain of the spike protein, i.e., RBD-Ig, and not a specific antibody and thus is less likely that the virus will develop escape variants that will escape the RBD-Ig. We now mention this in the discussion section.

We explored higher doses in vivo but the survival percentages were not improved. We now mention this in the results section as data not shown. We suggest that RBD-Ig reached saturation levels when using 75ug/mouse.

Comment: The observation that the RBD was more effective than the soluble ACE2 molecule was interesting and a possible explanation is provided (that increased virus proliferation outpaces the ACE2 dimer reagent), but the evidence to support this explanation was very limited and other potential explanations also exist that were not explored, for example reagent stability; and ability of the reagent to bind to bind via both arms of the dimer may be easier for RBD dimer binding to cellular ACE2 than ACE2 dimer binding to virus RBD.

Answer: We added an experiment in which we tested reagent stability, as can be seen in figure 4C, ACE2-Ig was even more stable than RBD-Ig. Please see results section.

In addition, the affinities of both reagents were already determined by Lui et al (please see reference: Lui I, Zhou XX, Lim SA, Elledge SK, Solomon P, Rettko NJ, et al. Trimeric SARS-CoV-2 Spike interacts with dimeric ACE2 with limited intra-Spike avidity. bioRxiv. 2020; 2020.05.21.109157. doi:10.1101/2020.05.21.109157). We discuss this in the discussion section. We agree with the reviewer that it may be easier for RBD-Ig dimer to bind to cellular ACE2 as compared to the ACE2-Ig dimer binding to virus RBD as the majority of RBDs are in an ACE2-inaccessible, “down” conformation, which requires opening for binding to ACE2 as suggested by Lui et al. We now added this explanation to the discussion section.

Comment: Furthermore, if the reagent worked well in the first place, then there should not be increased virus proliferation. Regardless, the observations in this study seem incremental over what is already known.

Answer: We added an experiment in which we checked virus titers at 5 days post infection in the lungs of infected mice treated with either RBD-Ig or ACE2-Ig. New figure 4D shows that RBD-Ig treated mice had lower virus titers in the lung as compared to ACE2-Ig treated mice. We now describe these results in the discussion section.

As mentioned above as far as we know it has not been previously shown that RBD-Ig can block interaction in-vivo and RBD-Ig was only use as a vaccine and not as therapeutic agent as we show here. Furthermore, the existing therapies such as highly potent antibodies target the spike protein, which is constantly mutating, as we constantly observe with the various variants, making such treatments inefficient. We now mention this in the introduction and discussion sections.

Reviewer #2:

Comment: The manuscript from Chaouat and Achdout describes the role of SARS-CoV-2 RBD or ACE2 proteins fused to Ig domain as a possible treatment against SARS-CoV-2. The authors characterize both these fusion proteins for binding via flow cytometry to either Spike or ACE2-expressing cell lines, in plaque reduction neutralization assays (PRNT) and in in vivo humanized ACE2 mouse model. They also try to understand the mechanisms of inhibition by RBD-Ig or ACE2-Ig to determine why RBD-Ig is better at neutralization compared to ACE2-Ig but unfortunately are unable to firmly determine the precise mechanism. The manuscript could be improved by providing more controls, average values with errors rather than one representative value (example) or determining if differential affinities between the RBD-Ig and ACE2-Ig is a reason for the higher neutralization potency of the former.

Answer: We thank the reviewer for the critical reading of our manuscript. We have responded to all comments and amended the manuscript accordingly (changes are in track changes). We have added all required controls as detailed below by the reviewer. In addition, the affinities of both reagents were already determined by Lui et al. (please see reference: Lui I, Zhou XX, Lim SA, Elledge SK, Solomon P, Rettko NJ, et al. Trimeric SARS-CoV-2 Spike interacts with dimeric ACE2 with limited intra-Spike avidity. bioRxiv. 2020; 2020.05.21.109157. doi:10.1101/2020.05.21.109157). We discuss this as well in the discussion section.

Comment: 1. Figure 1. In general, the manuscript defaults in showing one representative result without presenting the average values and errors from repeat experiments. In addition, it would be appropriate to include more controls such as non-specific spike expression, isotype controls or a different human receptor.

Answer: We now added more controls, i.e. background for each FACS staining and 293T cells overexpressing another human receptor: NKp46. Please see figure 2. In addition, we added another subfigure summarizing all staining experiments. Please see figure 2 and results section.

Comment: a. For Figure 1a-c, please include results from the other two representative experiments in terms of a column graph showing MFIs. This would provide an ability to show reproducibility between repeats.

Answer: We now added new figure summarizing the MFIs results of several experiments. Please see figure 2 and results section.

Comment: b. For 1a, is there another cell line expressing a non-relevant human receptor in addition to 293-T ACE2 that could be used that would show that RBD-Ig would be specific to 293T-ACE2? Can the authors please add an isotype control to show that the staining is specific to anti-Flag and not to secondary antibody?

Answer: We now added background (secondary only staining) for each cell line in figure 2A and in addition added the requested control; 293T cells overexpressing another human receptor: NKp46 demonstrating the staining is specific. The same was done for 2B, showing that RBD-Ig staining is specific to 293T-ACE2. Please see figure 2 and results section.

Comment: c. For 1b, can the authors use another cell line expressing mouse ACE2 or another non-relevant human receptor to show that RBD-Ig is specific to 293T-ACE2? Also would be good to use an isotype control or a RBD to another coronavirus that does not bind to 293T-ACE2?

Answer: We now added background (secondary only) for each cell line in figure 2B and 293T cells overexpressing another human receptor: NKp46 and indeed the staining of RBD-Ig is specific to 293T-ACE2. Please see figure 2 and results section.

Comment: d. For 1d, please see comments for 1a and 1b.

Answer: MFI’s and staining background were added in figure 2C and 2D. Please see figure 2 and results section.

Comment: 2. For Figure 2c and 2d, can the authors please provide the PRNT IC50 values for all three experiments in terms of nM (vs ug/ml). This would allow the comparison of the neutralization potency between ACE2-Ig and RBD-Ig.

Answer: We thank the reviewer for this important comment. As suggested figure 3C was changed and we now combined the values for all 3 experiments in terms of nmol/well. IC50 was added as requested. As can be seen both treatments inhibited SARS-CoV-2 to approximately the same extent. Please see results section.

Comment: 3. For Fig. 3, is it possible to also show the viral load data in terms of TCID50 or RT-qPCR to complement the weight loss data? Or any histopathological data?

Answer: We added an experiment in which we evaluated virus titers at 5 days post infection in the lungs of infected mice treated with either RBD-Ig or ACE2-Ig. New figure 4D shows that RBD-Ig treated mice had lower virus titers in the lung as compared to ACE2-Ig treated mice. Please see results section.

Comment: 4. One major possibility to explain the increased potency of RBD-Ig vs ACE2-Ig is that RBD-Ig has higher binding affinity to ACE2 compared to ACE2-Ig binding to RBD.
a. Can the authors provide any evidence of this using biophysical assays such as surface plasmon resonance or bio-layer interferometry?

Answer: Lui et al already tested the binding affinity and kinetics of different multimeric forms of recombinant ACE2 and Spike-RBD domain including ACE2-Fc and RBD-Fc. We now discuss this option in the discussion section.

Comment: b. If affinity is a potential mechanism, this could be formally tested by using a RBD variant with the 501Y mutation which shows increased affinity to human/mouse ACE2 and could be a very powerful experiment to show development of an improved therapeutic.

Answer: This is indeed a good suggestion; we are currently working on it. However, it will require a long time to complete these experiments as we need to repeat all in vitro and in vivo experiments. We thus prefer not to include this data in the current manuscript.

Comment: 5. Discussion: In general, the Discussion is lacking in direct and more thorough comparisons between previously published work (Huang et al, EMBO and Iwanaga et al). A deeper comparison would be helpful to understand the differences in potency or in vivo protection.
6. While these are ACE2 decoys, a discussion of Linsky et al, Science 2020 and Tanaka et al, bioarchive 2021 (doi: https://doi.org/10.1101/2021.03.09.434641) would also be appropriate in the Discussion. In particular, if there are insights to what drives neutralization mechanisms.

Answer: Comparisons between previously published works was added to discussion as requested.
