# Supplementary material for: SARS-CoV-2 receptor binding domain fusion protein efficiently neutralizes virus infection
Source: PLoS Pathog. 2021 Dec 20;17(12):e1010175. doi: 10.1371/journal.ppat.1010175 (PMC8722722; doi:10.1371/journal.ppat.1010175)
Supplement: Attachment — Submitted filename: pbp.docx [file ppat.1010175.s002.docx]

**Part I - Summary**

Reviewer #1:

Comment: The authors state that they are unaware of other studies that have used RBD-Fc to block in vivo. They are correct in that RBD-Fc may not have been used to block infection in vivo, prior to their study, but soluble RBD protein has been used, in Syrian hamster model (Zharadnik et al 2021, Nature Microbiology) - (previously published in BioRXiv in January 2021). Admittedly this was a higher affinity RBD, but on the other hand, it was a monomer, not a dimer which would limit its affinity. This is still a relevant point in the context of the current study.

Answer: We thank the reviewer for pointing out this recently published article which is very interesting and indeed is relevant to our research. We now mention this in the discussion pages 16-17. Their proposed drug has a high affinity but they did not show how long the drug remains in the lungs and whether it affects viral loads or mice survival.

Comment: Furthermore, another paper published this year has also shown that RBD-Fc can inhibit infection in vivo (Shin et al 2021. Int.J.Biol.Sci).

Answer: Shin et al used the same treatment as we, but we compare it to ACE2-Ig. Furthermore, the authors of this paper used regular mice, which as far as we and others know, cannot be infected with SARS-CoV-2. In contrast, we used humanized ACE2 mice. Thus we prefer not to cite this paper.

*(****1.*** *Letko, M., Marzi, A. & Munster, V. Functional assessment of cell entry and receptor usage for SARS-CoV-2 and other lineage B betacoronaviruses. Nat. Microbiol. 5, 562–569 (2020).* ***2.*** *Wan, Y., Shang, J., Graham, R., Baric, R. S. & Li, F. Receptor recognition by novel coronavirus from Wuhan: an analysis based on decade-long structural studies of SARS. J. Virol. https://doi.org/10.1128/jvi.00127-20 (2020).* ***3.*** *Winkler, E.S., Bailey, A.L., Kafai, N.M. et al. SARS-CoV-2 infection of human ACE2-transgenic mice causes severe lung inflammation and impaired function. Nat Immunol 21, 1327–1335 (2020). https://doi.org/10.1038/s41590-020-0778-2).*

Comment: I agree with the authors that a soluble RBD reagent may in theory have a better chance of preventing escape from variants, compared to some therapeutic antibodies, - this is a good idea!. However, my concern is that in this study, this RBD-Ig tool does not appear to be a very effective therapeutic in the first place, at least in the mouse model that has been used. It showed a moderate improvement in mouse survival (50% surviving rather than 20%) and a moderate ~50% reduction in PFU/lung. For these reason, I do not see that this tool is likely to be an improvement over the many different monoclonal antibody therapeutics that are in use, or in development, some of which are effective against a range of variants. Unfortunately this reduces the impact of this study.

Answer: We still think our proposed treatment as opposed to the available monoclonal antibody have potential as it targets ACE2, which is not mutating as opposed to the spike protein which constantly mutates. Furthermore, monoclonal antibodies drive immune escape through conformational changes of the spike protein.

Reviewer #2:

Comment: The authors have removed results from all of Figure 1. As such, Figure 1 is just a summary image, perhaps useful to just consolidate Figure 1 and 2 together.

Answer: Thank you, we combined the figures.
